# The effects of acute alcohol intoxication on the cognitive mechanisms underlying false facial recognition

**DOI:** 10.1007/s00213-016-4263-4

**Published:** 2016-03-15

**Authors:** Melissa F. Colloff, Heather D. Flowe

**Affiliations:** Department of Psychology, University of Warwick, Coventry, CV4 7AL UK; School of Sport, Exercise and Health Sciences, Loughborough University, Leicestershire, LE11 3TU UK

**Keywords:** Alcohol myopia theory, Face recognition, Accuracy, Response bias

## Abstract

**Rationale:**

False face recognition rates are sometimes higher when faces are learned while under the influence of alcohol. Alcohol myopia theory (AMT) proposes that acute alcohol intoxication during face learning causes people to attend to only the most salient features of a face, impairing the encoding of less salient facial features. Yet, there is currently no direct evidence to support this claim.

**Objectives:**

Our objective was to test whether acute alcohol intoxication impairs face learning by causing subjects to attend to a salient (i.e., distinctive) facial feature over other facial features, as per AMT.

**Methods:**

We employed a balanced placebo design (*N* = 100). Subjects in the alcohol group were dosed to achieve a blood alcohol concentration (BAC) of 0.06 %, whereas the no alcohol group consumed tonic water. Alcohol expectancy was controlled. Subjects studied faces with or without a distinctive feature (e.g., scar, piercing). An old-new recognition test followed. Some of the test faces were “old” (i.e., previously studied), and some were “new” (i.e., not previously studied). We varied whether the new test faces had a previously studied distinctive feature versus other familiar characteristics.

**Results:**

Intoxicated and sober recognition accuracy was comparable, but subjects in the alcohol group made more positive identifications overall compared to the no alcohol group.

**Conclusions:**

The results are not in keeping with AMT. Rather, a more general cognitive mechanism appears to underlie false face recognition in intoxicated subjects. Specifically, acute alcohol intoxication during face learning results in more liberal choosing, perhaps because of an increased reliance on familiarity.

There is a general consensus that alcohol impairs memory. Ninety-six percent of potential jurors, for example, agreed that intoxication reduces an eyewitness’s ability to recall persons and events (Benton et al. 2006). We know that blackouts—en bloc losses of memory—occur when blood alcohol concentration (BAC) rises rapidly, typically at concentrations above 0.20 % (Perry et al. 2006). Yet, an increasing body of literature now illustrates that alcohol can have no effects, or even beneficial effects, on memory (e.g., Colflesh and Wiley 2013; Mintzer and Griffiths 2001). Laboratory studies, which typically examine BACs in the range of 0.03 to 0.08 %, indicate that, at these doses, the influence of alcohol on memory depends on the cognitive functions required by the particular experimental task (e.g., Bisby et al. 2010; Söderlund et al. 2005). This study investigates the influence of acute alcohol intoxication on face recognition and in particular, the influence of intoxication on attention during encoding.

Alcohol myopia theory (AMT) is a widely accepted account of the cognitive effects of intoxication. This attention-allocation model posits that alcohol reduces the cognitive capacity available for controlled, effortful processing, which results in a state of disproportionate attention to salient stimuli, at the expense of weaker, peripheral cues (Steele and Josephs 1990). Indeed, the disparities between sober and intoxicated attention-allocation are well documented. Alcohol (*M* BAC = 0.06 %, in comparison to *M* BAC = 0.04 % or *M* BAC = 0 %) hinders the ability to attend to global information, unless the global form has been made salient (Lamb and Robertson 1987). Intoxicated subjects (*M* BAC = 0.06 %) make more fixations on salient items while neglecting peripheral information (Harvey et al. 2013a) and are less likely to notice an unexpected object in their visual field (*M* BAC = 0.04 %; Clifasefi et al. 2006).

Other research has investigated the memory deficits arising from alcohol’s myopic effect on attention. In an early study, subjects watched a brief staged event (a theft) and were interviewed immediately and 1 week later. At both time points, those who had consumed alcohol (*M* BAC = 0.10 %) freely recalled significantly less accurate information about what had happened during the theft (e.g., the location of the event, details about stolen objects) than the control or placebo subjects (Yuille and Tollestrup 1990). In a similar study, Van Oorsouw and Merckelbach (2012) asked bar patrons to watch a video of a mock crime from a perpetrator’s perspective. Four days later, the researchers asked subjects to give a detailed written description of the location, surroundings, and stolen objects in the video. Van Oorsouw and Merckelbach also found that previously moderately (*M* BAC = 0.06 %) and highly (*M* BAC = 0.17 %) intoxicated bar patrons were significantly less complete in recollecting the event than sober controls.

Specifically, though, AMT predicts that intoxicated individuals would exhibit impaired retrieval of peripheral, but not central, information. Indeed, this is what Schreiber Compo et al. (2011) found. Subjects spent almost an hour in a “barlab” (i.e., a room equipped with bar furniture and paraphernalia) and interacted with a “bartender”. Immediately after, subjects were asked to write down as much information as possible about their experience in the barlab. As predicted by AMT, there were no differences in the number of accurate central details (about the bartender) freely recalled, yet subjects in the alcohol group (*M* BAC = 0.07 %) freely recalled fewer accurate peripheral details (about the bar) than the placebo (*M* BAC = 0.01 %) and control subjects. But, does alcohol myopia affect face recognition?

Some research on alcohol intoxication and face recognition is in line with this possibility. In Yuille and Tollestrup’s (1990) study, subjects were also asked to attempt to recognize the thief when they were interviewed 1 week after viewing the staged event. Previously intoxicated subjects performed comparably to sober subjects when they were presented with a photo array that contained the target face. However, when the target face was not in the photo array, there was a tendency for previously intoxicated subjects to incorrectly pick a face. A similar pattern of results was evident in a field study in which subjects attempted to recognize a confederate with whom they had spoken 12 min earlier (Dysart et al. 2002). Subjects were presented with a single photograph. When the photo was the confederate, BAC was not significantly related to the correct identification rate, but when the photo was not the confederate, highly intoxicated bar patrons (*M* BAC = 0.09 %) were significantly more likely to make a false identification than minimally intoxicated bar patrons (*M* BAC = 0.02 %). In keeping with AMT, Dysart et al. hypothesized that intoxicated individuals only encoded the salient cues from the target face and then subsequently tried to match these with the salient cues on the test face. When the test face *was* the target, this strategy worked effectively. However, when the test face *was not* the target, the strategy resulted in a high number of false alarms. In short, the authors suggested that intoxicated subjects failed to encode the more subtle facial cues and, thus, had difficulty discriminating between similar-looking faces.

Other studies, however, have found no differences between sober and intoxicated face recognition ability. In a study by Hagsand et al. (2013), subjects watched a video of a mock kidnapping. Seven days later, subjects attempted to recognize the culprit from a photo array that either did or did not contain the target face. On both types of photo array, previously highly intoxicated (*M* BAC = 0.06 %), moderately intoxicated (*M* BAC = 0.04 %), and sober subjects all performed comparably. Harvey et al. (2013b) conducted a similar study in which subjects watched a slide sequence of a man stealing a mobile phone and then, 24 h later, attempted to recognize the culprit from a photo array that either did or did not contain the target face. Again, the authors found that previously intoxicated subjects (*M* BAC = 0.11 %) performed similarly to those who had been sober during encoding.

Given the mixed findings, our primary aim was to directly test whether intoxicated individuals differentially process faces during encoding in line with AMT. Namely, we examined whether acute alcohol intoxication during encoding causes people to attend only to the most salient features of a face. To this end, we followed Knapp and colleagues (2006) and manipulated the presence of distinctive facial features (scars, moles, piercings, tattoos, black eyes).

First, let us consider how distinctive features might impact on recognition performance when the learner is not intoxicated. Faces with distinctive features enjoy better recognition performance than faces without: the hybrid-similarity model (H-S model) can explain why (Knapp et al. 2006; Nosofsky and Zaki 2003; Zarkadi et al. 2009). Individual exemplars of study items are encoded and stored in memory. Subsequent recognition judgments are defined by global familiarity: the overall similarity between a test item and the exemplars stored in memory. The presence of a distinctive feature increases the number of matching features a test face and an exemplar share, which boosts their global familiarity and results in a high hit rate (HR). The addition of a target’s distinctive feature to a lure highlights that the lure mismatches the other exemplars in terms of this feature. This decreases their global familiarity and results in a low false alarm rate (FAR).

We wondered how alcohol intoxication might change these patterns in recognition performance. If intoxicated individuals do differentially process faces during encoding in line with AMT, then alcohol would serve to impair the distinctiveness advantage. Attention would be allocated to a salient distinctive feature at the expense of encoding other facial features. Therefore, subjects who were intoxicated during encoding should have a high FAR to faces with a distinctive feature that has previously been seen on another face.

However, if intoxicated individuals do not differentially process faces during encoding in line with AMT, then there are at least two other patterns of results that could be predicted using the H-S model and the existing intoxication literature. First, the pattern of recognition results found by Dysart et al. (2002) and Yuille and Tollestrup (1990) may have been because intoxicated individuals were more likely to make a positive recognition decision than their sober counterparts (Memon et al. 2003). This notion is concordant with studies illustrating that intoxicated subjects provide more subjective and erroneous information, while placebo subjects provide more “uncertain” responses (Schreiber Compo et al. 2011; Van Oorsouw and Merckelbach 2012). Put simply, it is possible that intoxicated individuals have a more liberal response criterion at test. If alcohol results in the adoption of a more liberal response bias, this would serve to reduce the amount of memorial information (i.e., global familiarity) required before a positive identification is made. Therefore, subjects who were intoxicated during encoding may have a higher HR and FAR to both distinctive and non-distinctive faces than those who were sober.

Second, Yuille and Tollestrup (1990) and Van Oorsouw and Merckelbach (2012) found that intoxicated subjects reported less information about an event than sober subjects. This pattern of results may have been because the intoxicated individuals encoded less information than their sober counterparts. If alcohol reduces the amount of information that is encoded, this would serve to decrease global familiarity. Therefore, subjects who were intoxicated during encoding may have a lower HR and FAR to both distinctive and non-distinctive faces than those who were sober.^1^

## Present study: predictions and controls

In the present study, subjects in the alcohol group were dosed to achieve a BAC of 0.06 %, whereas the no alcohol group consumed tonic water. Subjects studied faces with and without a distinctive feature. An old-new recognition test followed. Some of the test faces were “old” (i.e., had been previously studied), and the rest were “new” (i.e., had not been previously studied).

We had several different types of new faces. First, following Knapp et al. (2006), we had *unfamiliar distinctive* lures, which were novel faces that were not presented during the study phase but had a previously seen distinctive feature. Second, we had *unfamiliar non-distinctive* lures, which were novel faces that did not have a distinctive feature. We also had an additional two types of familiar face lures. *Familiar but no longer distinctive* lures were distinctive faces that were presented at study but had their distinctive feature removed at test. *Familiar but now distinctive* lures were non-distinctive faces that were presented at study but had a previously seen distinctive feature added at test. This design enabled us to examine the relative contribution of familiar distinctive features versus other familiar characteristics of faces on recognition decisions.

We expected to find the following pattern of results. First, according to the H-S model, distinctive faces should be better remembered than non-distinctive faces. Those who consume tonic should have a higher HR to distinctive faces, compared to non-distinctive faces. They should also have a lower FAR to unfamiliar distinctive lures than to unfamiliar non-distinctive lures. Second, if alcohol causes people to focus on the most salient features during encoding as per AMT, then subjects in the alcohol compared to the no alcohol group should identify test faces as “old” more often if they have a familiar distinctive feature. Those who consume alcohol should have a higher HR to distinctive faces, compared to non-distinctive faces. They should also have a higher FAR to unfamiliar distinctive lures than to unfamiliar non-distinctive lures.

We also instituted a number of controls to isolate alcohol’s effects on recognition processes. First, we manipulated whether the test session was immediate or delayed (24 h), to confirm that any differences in performance were not simply due to subjects being intoxicated or sober at retrieval. It seems that alcohol impairs encoding more than retrieval (Birnbaum et al. 1978); however, encoding and retrieval both often take place while the subject is intoxicated (Dysart et al. 2002; Schreiber Compo et al. 2011). Second, we used a balanced placebo design—in which alcohol administration was crossed with the expectancy of receiving alcohol—to confirm that any effects of alcohol on facial recognition were due to the physiological action of the drug. The expectancy of alcohol can cause or potentiate alcohol-induced memory impairments (Assefi and Garry 2003); however, face recognition studies have not yet disentangled the possible psychological and physiological effects.

Finally, if alcohol causes people to rely on familiar distinctive features more than other aspects of the face, then subjects in the alcohol condition should be prone to identifying test faces as “old” when they also have familiar distinctive features. That is, those who have consumed alcohol should have a higher FAR to familiar but now distinctive lures than familiar but no longer distinctive lures. Conversely, if sober subjects rely on familiar distinctive features to a lesser extent, then the FAR will be similar for both lure types.

## Method

### Design

We used a 2 × 2 × 2 × 2 × 3 mixed experimental design. Beverage administered (no alcohol, alcohol), beverage expected (no alcohol, alcohol), and test session (immediate, delayed) were manipulated between subjects. Study faces (distinctive feature, no distinctive feature) and test faces (match, face varies, feature varies) were manipulated within subjects. The research was approved by the University of Leicester’s Ethics Committee.

### Subjects

One hundred females (aged 18–32, *M* = 20.55, SD = 2.30 years) participated in the study. There were 9 to 17 subjects in each of the between-subjects conditions. Subjects were recruited from the University of Leicester via posters and electronic advertisements. Prior to arrival at the laboratory, subjects were pre-screened. Individuals with medical conditions or those who scored over 10 on the Alcohol Use Disorders Identification Test (Babor et al. 2001) were unable to participate. Those who were eligible received a small payment of between £10 and £20.

### Apparatus and materials

In accordance with other research, the face stimuli were developed using 80 photographs from The Florida Department of Corrections Inmate Database (Colloff et al., submitted for publication; Flowe et al. 2014; Zarkadi et al. 2009). The selected photographs depicted males between 18 and 24 years old, with short brown hair, and no distinctive features. As previous research indicates that race (Hilliar et al. 2010), gender (Ward et al. 2012), and emotional expression (Flowe et al. 2014; Flowe 2012) may influence cognitive processes, photographs depicted white males, exhibiting neutral expressions, facing directly towards the camera. Using Adobe Photoshop CS5, the photographs were normalized. They were changed to grayscale, and the backgrounds were removed.

**Fig. 1:**
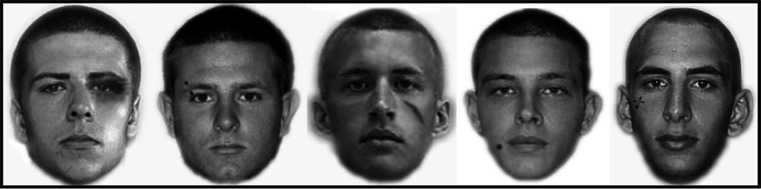
Examples of faces with digitally added distinctive features (from *left* to *right*: a black eye, an eyebrow piercing, a scar, a mole, a tattoo)

We randomly selected 60 faces to serve as the study faces, and 30 of these study faces were randomly selected to be the distinctive study set. Following Knapp et al. (2006), we digitally added a distinctive feature to these faces. A range of features were added to ensure that the semantic content of the features were not confounded with fixation biases to particular screen locations (see Fig. 1). The remaining 30 study faces became the non-distinctive study set.

**Fig. 2:**
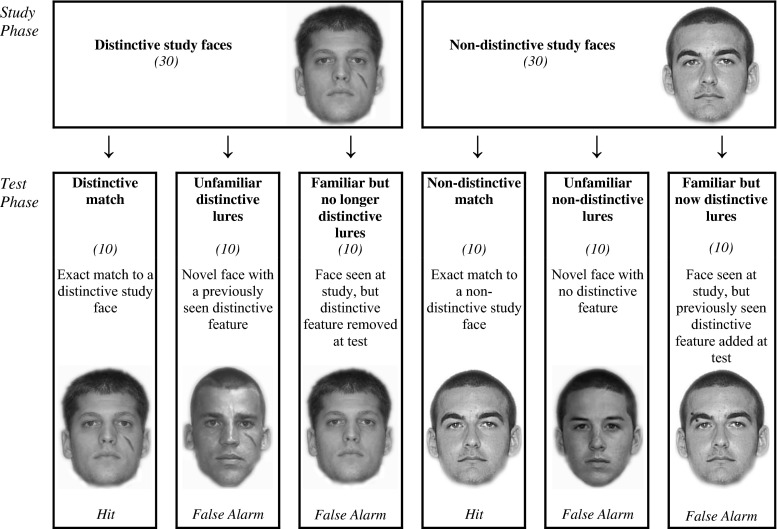
Composition of the study and test phase in the face recognition task. The values in *parentheses* indicate the number of trials conducted for each face type. *Hit* indicates that if the subject states they have seen this face before, it is a correct recognition decision. *False alarm* indicates that if the subject states that they have seen this face before, it is an incorrect recognition decision

The test phase consisted of six different types of faces (10 of each). There were two types of “old” faces that had been seen during the study phase: (1) faces that were an exact match to the distinctive study faces (*distinctive match*) and (2) faces that were an exact match to the non-distinctive study faces (*non-distinctive match*). There were two types of unfamiliar faces: (3) novel faces that were not presented during the study phase but had a previously seen distinctive feature (unfamiliar distinctive lures) and (4) novel faces that did not have a distinctive feature (unfamiliar non-distinctive lures). Finally, there were two types of familiar faces: (5) faces that were presented at study but had their distinctive feature removed at test (familiar but no longer distinctive lures) and (6) faces that were presented at study but had a distinctive feature added at test (familiar but now distinctive lures). Figure 2 shows the composition of the study and test phase.

Intoxication levels were measured by breath samples using an AlcoHAWK^TM^ Slim. The breathalyzer converts breath alcohol ratio into BAC.

### Procedure

In an attempt to match the absorption rate of alcohol, subjects avoided eating for 4 h prior to the experiment. Subjects were tested individually. At the start of the testing session, a pregnancy test was administered to ensure the subject was not pregnant, and her height and weight were measured for purposes of dosing. A baseline breath sample was also taken to ensure the subject had a BAC of 0.00 % at the start of the study.

Subjects in the alcohol condition received three cups containing a mixture of vodka (37.5 %) and tonic water in a 1:5 ratio. The BAC of individuals receiving alcohol was intended to be 0.06 % on average, which is equivalent to 0.60 g/L or 0.57 g/kg. We chose this BAC level for two main reasons. First, attention-allocation disruptions have previously been observed at this level of intoxication (Harvey et al. 2013a; Lamb and Robertson 1987) or lower (Clifasefi et al. 2006). Second, like the majority of studies that have administered alcohol, we did not want subjects’ BACs to exceed 0.08 %, for ethical reasons. The dose of vodka required to produce the target peak BAC was computed separately for each subject by using her height and weight (see Curtin and Fairchild 2003). The amount of alcohol administered was 101.86 ml (SD = 27.77 ml), on average. Subjects in the no alcohol condition received three cups containing tonic water. The quantity of tonic water was equivalent to the total amount of liquid the subject would have received in the alcohol condition. To disguise the beverage content, we followed previous research and put vodka soaked limes in each drink and rimmed each cup with vodka (Assefi and Garry 2003). All drinks were prepared in a separate room away from the subject.

We manipulated subjects’ alcohol expectancies using procedures that have been successful in previous research (e.g., Craig et al. 2009). We clearly labeled the cups as “Vodka & Tonic” or “Tonic Water” and verbally informed the subject that her drinks either did or did not contain alcohol, depending on the expectancy condition to which she had been assigned. Those who were told that their drinks contained alcohol were not given any specific information about the dose that they had ostensibly received. A researcher who was blind to the content of the beverages administered the drinks.

To maintain a steady ingestion pace, subjects consumed each drink within 5 min. After a further 15 min (30 min after drinking began), subjects’ BACs were recorded. We told subjects that we used a standardized procedure, and so they would be repeatedly breathalyzed regardless of what drink they had consumed.

Next, subjects were escorted into a separate room to complete the face recognition task. During the *study phase*, faces were presented in the center of a computer screen (size, 10 cm × 10 cm; duration, 3 s), in a randomly generated order using E-Prime software. Subjects were instructed that they should attempt to remember the faces, as they would be tested on them later. Subjects in the immediate testing condition completed a 5 min anagram filler task before the *test phase* commenced. Those in the delayed testing condition were emailed a link and completed the test phase at home 24 h later, when sober. During the *test phase*, subjects were instructed to indicate whether they had previously seen each face and rate their confidence in their decision on a single 20-point Likert scale, ranging from 1 (*new face*, *extremely confident*) to 10 (*new face*, *not at all confident*) and 11 (*old face*, *not at all confident*) to 20 (*old face*, *extremely confident*). They were informed that a face was “old” if it was exactly the same as a study face; it was “new” if it differed in any way from the study face. Subjects were provided with pairs of example study and test faces to ensure that they understood which test faces were “old” and which were “new”. Each example pair was clearly labeled with the correct answer. The faces and distinctive features used as examples had not been used in the study phase and were not seen again once the test phase began.

On completion, subjects were asked what drink they thought they had consumed. Those who had consumed alcohol were only released from the study when their BAC was below 0.02 %. All subjects remained in the laboratory for at least 2 h to make it more difficult for them to guess which drink they had received.

### Statistical analyses and derivation of measures

We computed the proportion of positive identifications each subject made to the six different test face types. A positive identification is when a subject stated a face was “old”.

First, following Knapp et al. (2006), we examined the hits and false alarms made to distinctive and non-distinctive faces in each of our experimental conditions. We conducted a 2 (beverage administered) × 2 (beverage expected) × 2 (test session) × 2 (face type) × 2 (target) mixed ANOVA on subjects’ hit and false alarms, with face type (distinctive vs. non-distinctive) and target (present vs. absent) as the within-subjects factors. Hits to distinctive and non-distinctive faces were positive identifications to distinctive match and non-distinctive match faces, respectively. False alarms to distinctive and non-distinctive faces were positive identifications to unfamiliar distinctive lures and unfamiliar non-distinctive lures, respectively.

Next, we constructed a confidence-based receiver operating characteristic (ROC) plot using the hits and false alarms made to distinctive and non-distinctive faces by subjects in the two beverage-administered conditions. Again, hits to distinctive and non-distinctive faces were positive identifications to distinctive match and non-distinctive match faces, respectively. False alarms to distinctive and non-distinctive faces were positive identifications to unfamiliar distinctive lures and unfamiliar non-distinctive lures, respectively.

Finally, we compared the false alarms made to the two familiar lure types (familiar but no longer distinctive lures and familiar but now distinctive lures) in each of our experimental conditions. We conducted a 2 (beverage administered) × 2 (beverage expected) × 2 (test session) × 2 (lure face type) mixed ANOVA, with the false alarm rate as the dependent variable. Wilks’ Lambda test statistic was used throughout. Cohen’s *d* effect sizes for repeated measured *t* tests were calculated using a correction for the correlation between the two groups.

## Results

### Manipulation check

Breathalyzer readings taken 30 min after the beginning of beverage consumption indicated that all subjects in the no alcohol group had a BAC of 0.00 %, while the BAC of subjects in alcohol group was significantly higher (*M* BAC = 0.06 %, 95 % CI [.05, .06], SD = 0.02, range 0.02–0.09 %), *t*(54) = 24.94, *p* < .001.

In those who consumed tonic, there was a significant association between the beverage expected and the drink subjects believed they had consumed, *χ*^2^ (1, *N* = 44) = 25.14, *p* < .001, *ϕ* = 0.76. Specifically, 73 % of those who were told their drinks were vodka and tonic believed that they had consumed alcohol, and 100 % of those who were told their drinks were tonic believed that they had not consumed alcohol. In those who consumed alcohol, there was a significant association between the beverage expected and the drink subjects believed they had consumed, *χ*^2^ (1, *N* = 55) = 9.65, *p* = .002, *ϕ* = 0.42. Specifically, 100 % of those who were told their drinks were vodka and tonic believed that they had consumed alcohol, but only 31 % of those who were told their drinks were tonic believed that they had not consumed alcohol.

### Distinctive and non-distinctive faces

#### Hits and false alarms

Recall that the H-S model predicts better recognition to distinctive faces, but AMT predicts that intoxication may increase the number of false alarms to unfamiliar distinctive lures. Subjects’ hit and false alarm rates for distinctive and non-distinctive faces across the experimental conditions are presented in Table 1. First, it is important to note that the mixed ANOVA indicated there was a main effect of target, *F*(1, 92) = 11.86, *p* = .001, *η*_p_^2^ = .11. Subjects were more likely to positively identify a face they had seen before (*M* = .48, 95 % CI [.45, .52]) than false alarm to a face they had not seen before (*M* = .42, 95 % CI [.39, .46]). This suggests that both sober and intoxicated subjects were able to perform the task proficiently.

**Table 1:** Means and standard deviations of subjects’ hit and false alarm rates to distinctive (D) and non-distinctive (ND) faces as a function of beverage administered, beverage expected, and test session

	Hit Rate				False Alarm Rate			
Condition	D		ND		D		ND	
	*M*	SD	*M*	SD	*M*	SD	*M*	SD
Administered alcohol								
Expected alcohol								
Immediate testing	.56	.24	.42	.20	.46	.21	.42	.18
Delayed testing	.68	.20	.49	.18	.63	.21	.40	.23
Expected tonic								
Immediate testing	.60	.21	.42	.20	.55	.22	.35	.19
Delayed testing	.52	.26	.32	.19	.50	.19	.37	.23
Administered tonic								
Expected alcohol								
Immediate testing	.58	.21	.41	.13	.39	.20	.36	.19
Delayed testing	.63	.15	.35	.26	.54	.20	.35	.14
Expected tonic								
Immediate testing	.54	.19	.34	.16	.39	.19	.28	.16
Delayed testing	.57	.18	.28	.20	.53	.19	.25	.19

*Note* False alarm rates to D and ND faces were calculated using positive identifications to *unfamiliar distinctive* lures and *unfamiliar non-distinctive* lures, respectively

Next, onto the predictions of the H-S model and AMT. The mixed ANOVA indicated that there was a main effect of face type, *F*(1, 92) = 96.89, *p* < .001, *η*_p_^*2*^ = .51. Subjects were more likely to positively identify distinctive (*M* = .54, 95 % CI [.51, .58]) than non-distinctive (*M* = .36, 95 % CI [.33, .39]) faces. However, this was qualified by a marginally significant face type × target interaction, *F*(1, 92) = 3.51, *p* = .06, *η*_p_^2^ = .04. We conducted four Bonferroni-corrected repeated measures *t* tests, with the target as the repeated factor. Results indicated that face type had a differential effect on positive identifications, depending on whether the target was present or absent. When the test face was distinctive, subjects were more likely to positively identify targets (*M* = .58, 95 % CI [.54, .63]) than lures (*M* = .50, 95 % CI [.46, .54]), *t*(99) = 3.85, *p* < .001, *d* = 0.38. When the test face was non-distinctive, subjects were not more likely to positively identify targets (*M* = .38, 95 % CI [.34, .42]) than lures (*M* = .35, 95 % CI [.31, .39]), *t*(99) = 1.30, *p* = .20, *d* = 0.13. Subjects made more correct positive identifications to distinctive faces than non-distinctive faces, *t*(99) = 9.01, *p* < .001, *d* = 0.87. They also made more incorrect positive identifications to distinctive faces than non-distinctive faces, *t*(99) = 6.86, *p* < .001, *d* = 0.67. Thus, taken together, this suggests that subjects responded more liberally to distinctive faces than non-distinctive faces, but they were also better able to discriminate between a target and a lure when the face was distinctive. In line with the predictions of the H-S model, distinctive faces were more accurately recognized than non-distinctive faces. But, did alcohol impair the distinctiveness advantage?

Interestingly, there was no face type × target × beverage administered interaction, *F*(1, 92) = 0.80, *p* > .250, *η*_p_^2^ = .01. Contrary to the predictions of AMT, this indicates that both sober and intoxicated subjects recognized distinctive faces better than non-distinctive faces. However, there was a main effect of beverage administered, *F*(1, 92) = 3.87, *p* = .05, *η*_p_^2^ = .04. Subjects who had consumed alcohol made more positive identifications (*M* = .48, 95 % CI [.44, .52]) than those who had consumed tonic (*M* = .42, 95 % CI [.38, .47]). This suggests that subjects who were intoxicated at encoding employed a more liberal response criterion than those who were sober.

Finally, did any of our controls modulate these effects? The beverage administered findings held regardless of test session, *F*(1, 92) = 0.03, *p* > .250, *η*_p_^2^ = .00, and the beverage expected, *F*(1, 92) = 0.00, *p* > .250, *η*_p_^2^ = .00. This suggests that the liberal responding was due to intoxication at encoding rather than retrieval and could not be induced by simply being told one had consumed alcohol. However, we did find a significant face type × test session interaction, *F*(1, 92) = 6.62, *p* = .012, *η*_p_^2^ = .07. After immediate testing, subjects were more likely to positively identify distinctive faces (*M* = .52, 95 % CI [.47, .57]) than non-distinctive faces (*M* = .38, 95 % CI [.34, .43]), *t*(51) = 5.94, *p* < .001, *d* = 0.87. After delayed testing, subjects were also more likely to positively identify distinctive faces (*M* = .57, 95 % CI [.52, .62]) than non-distinctive faces (*M* = .34, 95 % CI [.29, .39]); however, the distinctiveness effect was stronger after a delay, *t*(47) = 8.60, *p* < .001, *d* = 1.27.

So far, our results indicate that subjects responded more liberally to distinctive faces, but, in accordance with the H-S model, recognized distinctive faces more accurately than non-distinctive faces. We found no evidence that alcohol impaired this distinctiveness advantage. Instead, subjects who were intoxicated at encoding tended to respond more liberally than their sober counterparts.

#### Confidence-based ROC plot

To further confirm these findings, we constructed an ROC plot. ROC analysis is a theory-free technique that plots HR/FAR pairs over decreasing levels of confidence. Confidence is used as an indicator of subjects’ willingness to make a positive identification, with decreasing levels of confidence representing more liberal responding. In short, ROC analysis permits examination both of subjects’ ability to discriminate between faces they have and have not seen before, and their response bias  (Macmillan and Creelman 1991).

**Fig. 3:**
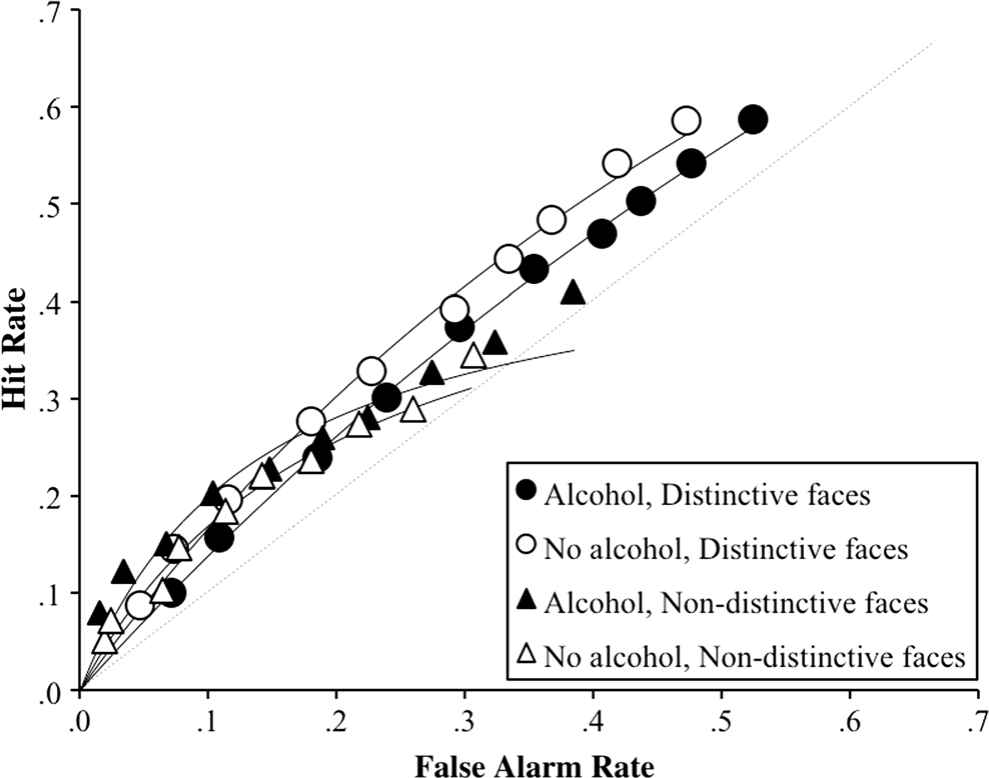
Confidence-based ROC curves for distinctive and non-distinctive faces in subjects who had and had not consumed alcohol at encoding

To construct our ROC curves, we collapsed the data across subjects within the same beverage-administered group. We used subjects’ confidence ratings to positive identification decisions (ratings 11–20 on the Likert scale), so that each curve would have 10 operating points of decreasing confidence (i.e., 20, 19, 18 etc.). Figure 3 shows the confidence-based ROC curves for distinctive and non-distinctive faces in subjects who had and had not consumed alcohol at encoding. On each curve, the HR/FAR pair plotted on the lower left was computed by calculating the proportion of hits and false alarms that were made with a confidence of 20. Moving to the right, the next HR/FAR pair was computed by calculating the proportion of hits and false alarms that were made with a confidence of 19 or higher. The cumulative hit and false alarm proportions were calculated in this manner across the rest of the curve. Thus, on each curve, the HR/FAR pair plotted on the farthest right is the cumulative hit and false alarm rates for all subjects across all 10 operating points.

First, it is clear from Fig. 3 that the ROC points for the distinctive faces have shifted more to the right than the ROC points for the non-distinctive faces. This shift indicates an increase in both hits and false alarms for distinctive faces. Again, as we found in our previous analyses, this suggests that subjects responded more liberally to distinctive faces than non-distinctive faces. What is also evident is that the ROC curves for the distinctive faces tend to fall further from the dashed chance line and closer to the top left corner of the plot than the ROC curves for the non-distinctive faces, and this is true for both beverage-administered groups. Again, in line with the predictions of the H-S model, but contrary to the predictions of AMT, this suggests that both sober and intoxicated subjects recognized distinctive faces better than non-distinctive faces. The astute reader may notice, however, that the ROC curve for the alcohol group does fall *slightly* below that of the non-alcohol group for distinctive faces. Discriminability appears lower when there is greater variability in criterion placement across subjects (Benjamin et al. 2009). Given the range of BAC in the alcohol group, and our finding that alcohol results in more liberal responding, it is possible that the ROC curve has been pulled down because of variable criterion placement by subjects at different levels of intoxication.

Finally, and perhaps most interestingly, it is also clear that, for both face types, the ROC curves for subjects who had consumed alcohol are shifted more to the right than the ROC curves for subjects who had not consumed alcohol. Again, in accordance with the previous analyses, this suggests that subjects who were intoxicated at encoding employed a more liberal response criterion than those who were sober.

### False alarms to familiar lures

**Fig. 4:**
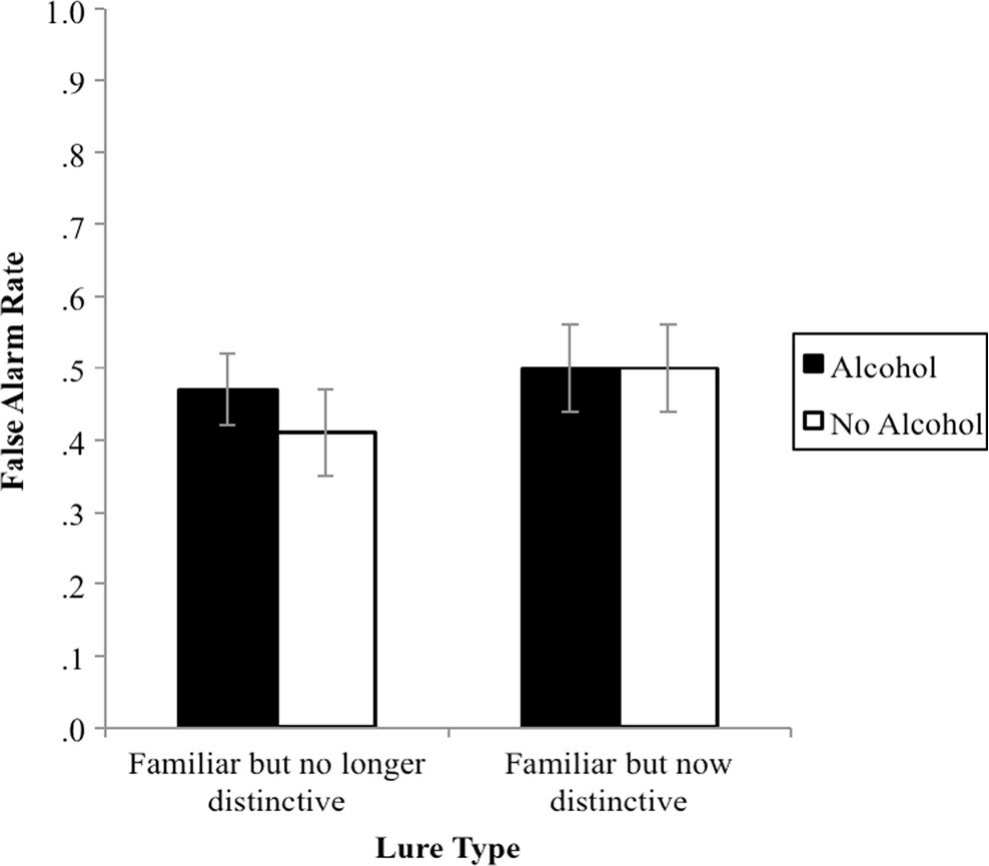
Mean false alarm rates to *familiar but no longer distinctive* lures and *familiar but now distinctive* lures, as a function of beverage administered. *Error bars* are 95 % CIs

In our final analysis, we investigated how intoxicated and sober subjects used familiar facial information. We examined the false alarms made to our two familiar face types to test whether subjects were more reliant on familiar distinctive features versus other familiar aspects of the faces during recognition and whether this was the case particularly for intoxicated subjects. Subjects’ false alarm rates for familiar but now distinctive lures and familiar but no longer distinctive lures as a function of beverage administered are presented in Fig. 4. The mixed ANOVA indicated a main effect of lure type, *F*(1, 92) = 5.12, *p* = .03, *η*_p_^2^ = .05. Subjects made more false alarms to familiar but now distinctive lures (*M* = .50, 95 % CI [.46, .55]), than to familiar but no longer distinctive lures (*M* = .44, 95 % CI [.40, .48]). This was not qualified by a lure type × beverage administered interaction, *F*(1, 92) = 1.17, *p* = .28, *η*_p_^2^ = .01, nor was there a main effect of beverage administered, *F*(1, 92) = 2.01, *p* = .16, *η*_p_^2^ = .02. This suggests that AMT cannot account for our results: both sober and intoxicated subjects picked familiar faces more often when those faces also had a familiar distinctive feature. However, it is clear from Fig. 4 that intoxicated subjects made a very high number of false alarms to both types of familiar lure faces.

Finally, there was a main effect of test session, *F*(1, 92) = 4.28, *p* = .04, *η*_p_^2^ = .04. Subjects who were tested after a delay made more false alarms (*M* = .51, 95 % CI [.46, .55]) than those who were tested immediately (*M* = .44, 95 % CI [.39, .48]). This suggests that, regardless of whether subjects were previously intoxicated or not, their ability to correctly reject a familiar face was worse after a 24-h delay. No other main or interaction effects were significant (all *F*s < 2.90, all *p*s > .09).

## Discussion

We asked whether intoxicated individuals differentially process faces during encoding in line with AMT. Our results indicated that both sober and intoxicated groups were better able to discriminate between targets and lures when faces had distinctive features, and both groups responded more liberally to distinctive faces. Subjects who were intoxicated at encoding responded more liberally at test compared to their sober counterparts. We will consider these findings in turn.

Taken together, our findings suggest that the H-S model applied to those who were sober and intoxicated at encoding, alike. In both groups, distinctive faces elicited better recognition performance than non-distinctive faces (Knapp et al. 2006; Nosofsky and Zaki 2003). It was not predicted a priori that distinctive faces would also elicit a higher FAR than non-distinctive faces, but careful consideration of our experimental task can help to explain this finding. Although the number of distinctive and non-distinctive faces seen by subjects was equal, we used only five distinctive features. The H-S model suggests that recognition judgments are defined by global familiarity: the overall similarity between a test item and the exemplars stored in memory. Because the distinctive feature on the lure also appeared on multiple exemplars, this could have increased global familiarity and resulted in a higher FAR to lures with distinctive features. The fact that there were more positive identifications to distinctive faces after a delay is inline with this notion: Even when memory had weakened, faces with distinctive features had a high global familiarity and so received more positive identifications than non-distinctive faces. More generally, this explanation is consistent with the idea of “cue overload” (Watkins and Watkins 1975). We know that the FAR is often higher to foils from categories from which more items have been studied (Gallo et al. 2004; Robinson and Roediger 1997; Shiffrin et al. 1995).

We also found that those who were intoxicated at encoding responded more liberally at test. Subjects’ proclivity to positively identify faces did not depend on whether they thought that they were intoxicated during encoding nor on whether they were intoxicated or sober at test. Thus, it seems that feeling the physiological effects of alcohol during encoding led people to adjust their response strategy. Other research has found an alcohol-linked increase in lure but not target identifications (Dysart et al. 2002; Yuille and Tollestrup 1990), whereas we found an increase in lure *and* target identifications. Nevertheless, both patterns could be due to intoxicated subjects using a more lax decision criterion. Under conditions in which subjects can successfully discriminate targets from lures, the false alarm rate will be affected to a greater extent than the hit rate as the criterion shifts to a more liberal position. However, under conditions in which the target is not particularly well remembered, a liberal shift in criterion placement can affect the hit rate to the same extent as the false alarm rate (Wickens 1942). Our task was arguably more difficult than previous studies because, for example, our subjects saw many similar-looking faces presently briefly, whereas subjects in previous studies only had to recognize one individual with whom they had watched or interacted with in person (Dysart et al. 2002; Yuille and Tollestrup 1990). Accordingly, memory accuracy was lower across the board in our study, and this could explain why we saw an increase in both hits and false alarms in our intoxicated subjects.

Interestingly, a liberal response criterion seems to be associated with an increase in familiarity processing (see Meissner et al. 2005 for a review). That is, people tend to base their decisions on a feeling that the face has previously been encountered, rather than retrieving specific contextual information about the face, such as source, time, and place (Yonelinas 2002). Other researchers have observed an increased reliance on familiarity in intoxicated subjects because of an impairment in recollection (Bisby et al. 2010; Curran and Hildebrandt 1999). Our results seem to bear this out: not only did our intoxicated subjects have a tendency to respond more liberally, they also had a high FAR to both types of familiar lures.

The influence of alcohol myopia on subsequent memory ability was not supported at our dosage levels. According to AMT, attention is allocated to the salient distinctive features at the expense of encoding holistic, global appearance (Dysart et al. 2002; Josephs and Steele 1990). Based on this logic, the FAR to novel faces with familiar distinctive features should have been particularly high in those who were intoxicated compared to those who were sober at encoding. One possibility is that for AMT to hold, the distinctive feature needed to be salient in both an “absolute” and a “relative” respect. In an absolute sense, our distinctive features are likely to have been “salient” compared to one’s previous experience of faces. However, half of the faces in our experiment had distinctive features, and therefore, the features may not have been considered to be “salient” relative to the other faces in the stimulus set. Indeed, studies have shown that the effects of “distinctiveness” can be contingent on what other items are included in the task (Hosie and Milne 1996).

### Limitations and future directions

Performance in our study is consistent with the levels of recognition accuracy reported by Knapp et al. (2006). However, it is apparent that subjects found the task difficult; subjects were equally likely to positively identify non-distinctive targets and lures. Future studies could employ an easier task, such as using fewer target faces and giving distinctive study faces unique distinctive features, to ensure that our findings are generalizable. However, we do not believe that poor performance overall has impacted upon our conclusions about AMT. AMT predicts that alcohol intoxication at encoding will impair subjects’ ability to discriminate between old and new distinctive faces. Subjects who were sober at encoding were able to recognize distinctive faces proficiently^2^ so there was certainly room for intoxicated subjects’ accuracy to fall below this. Interestingly, we found that subjects who were intoxicated at encoding were also able to recognize distinctive faces proficiently^3^. In short, the non-significant difference between sober and intoxicated individuals for the distinctive faces is unlikely to be due to floor effects.

Our findings add to the increasing number of studies that suggest that caution should be taken when applying AMT to face recognition performance when subjects are intoxicated to around the level of the legal driving limit (Hagsand et al. 2013; Harvey et al. 2013b). However, the risk of cognitive impairment increases with higher levels of intoxication (Bisby et al. 2010; Perry et al. 2006). Therefore, future research should test whether faces are differentially processed when intoxication levels are higher. Given ethical concerns about heavily dosing subjects in the lab, future research could test bar patrons who often self-intoxicate to greater levels (Dysart et al. 2002; van Oorsouw and Merckelbach 2012). In the field, one has no control over other factors, such as alcohol expectancy. However, while our expectancy manipulation was, on the whole, successful, subjects were generally aware when they had consumed alcohol. This is a common occurrence in lab research when BACs exceed 0.05 % (see Sayette et al. 1994, for a review). Therefore, despite concerns about ability to control other potentially interesting factors in the field, we believe that recruiting subjects who have self-intoxicated to greater levels is a worthy and necessary avenue for future research.

To conclude, we have extended past research by examining the cognitive processes underling alcohol-related face recognition performance. Intoxicated individuals did not seem to differentially process faces during encoding in line with AMT. They did, however, tend to respond more liberally at retrieval. This pattern may indicate an alcohol-induced increase in familiarity-based processing.

## Footnotes

1 We thank an anonymous reviewer for this suggestion.

2 For subjects who were sober at encoding, the hit rate to distinctive faces (*M* = .58, 95 % CI [.53, .64]) was significantly higher than the false alarm rate to unfamiliar distinctive lures (*M* = .47, 95 % CI [.41, .54]), *t* (43) = 3.26, *p* = .002.

3 For subjects who were intoxicated at encoding, the hit rate to distinctive faces (*M* = .59, 95 % CI [.52, .65]) was significantly higher than the false alarm rate to unfamiliar distinctive lures (*M* = .53, 95 % CI [.47, .58]), *t*(55) = 2.20, *p* = .032.
